# Local Antibiotic Delivery Systems in Diabetic Foot Osteomyelitis: A Brief Review

**DOI:** 10.1900/RDS.2021.17.75

**Published:** 2021-10-31

**Authors:** Christos Chatzipapas, Makrina Karaglani, Nikolaos Papanas, Konstantinos Tilkeridis, Georgios I. Drosos

**Affiliations:** 1 Department of Orthopaedic Surgery, Democritus University of Thrace, University Hospital of Alexandroupolis, Alexandroupolis, Greece; 2 Diabetes Centre - Diabetic Foot Clinic, Second Department of Internal Medicine, Democritus University of Thrace, University Hospital of Alexandroupolis, Alexandroupolis, Greece.

**Keywords:** diabetic foot osteomyelitis, local antibiotic delivery, PMMA, calcium sulfate

## Abstract

Diabetic foot osteomyelitis (DFO) is a severe, difficult to treat infection. Local antibiotic delivery has been studied as a potential therapeutic adjunct following surgery for DFO. This review aims to summarize the evidence on local antibiotic delivery systems in DFO. PubMed database was searched up to March 2020. Overall, 16 studies were identified and included: 3 randomized controlled trials (RCTs), 3 retrospective studies (RSs), and 10 case series. In the RCTs, gentamicin-impregnated collagen sponges significantly improved clinical healing rates and slightly improved duration of hospitalization. In the RSs, antibiotic-impregnated calcium sulfate beads non-significantly improved all healing parameters, but did not reduce post-operative amputation rates or time of healing. The majority of case series used calcium sulfate beads, achieving adequate rates of healing and eradication of infection. In conclusion, evidence for add-on local antibiotic delivery in DFO is still limited; more data are needed to assess this therapeutic measure.

## 1 Introduction

Diabetes mellitus increases the risk of foot infections, some cases of which progress to diabetic foot osteomyelitis (DFO) [1, 2]. Foot deformity, peripheral neuropathy, peripheral arterial disease, and minor injury increase the risk of diabetic foot lesions [2-5]. The development of biofilms in chronic wounds represents an additional challenge, since they protect pathogens from removal by host immunity and systemically administered antibiotics [6].

Management of DFO may be surgical or medical, depending on patient characteristics [7]. Surgery is especially useful in the event of pus, sequestrum, gangrene, or antibiotic-resistant bacteria [8]. Instead of amputation, debridement offers the advantage of removing necrotic while preserving healthy bones and tissues [9]. This approach is sometimes accompanied by local antibiotic delivery [3].

Local antibiotics offer the following advantages: higher local antibiotic concentration, longer duration, and fewer side effects [3]. At the same time, they act as a bone substitute that fills the dead space caused by bone resection [10]. Polymethylmethacrylate (PMMA) beads are the major representative of nonbiodegradable carriers [11]. Antibiotic release from PMMA beads is initially high during the first 48-72 hours, but quickly falls to lower levels, and may elute for weeks or even years [11]. Disadvantages include the high temperature it produces and the surgical removal of the beads required upon completion of drug release [12, 13].

During the last 2 decades, biodegradable carriers have been developed: proteins (collagen, gelatin, thrombin etc.), synthetic polymers, grafts, and substitutes (calcium sulfate or phosphate) [14]. These act as a matrix for new bone growth. During their degradation, additional release of antibiotics occurs, prolonging their action and preventing biofilm formation on their surface [15].

The aim of this brief review is to summarize the evidence on add-on local antibiotic delivery in the surgical management of DFO.

## 2 Search strategy

We performed a search in the PubMed database for studies published up to December 2020 on the management of patients with DFO using an implantable antibiotic delivery system. We excluded case reports, case series with fewer than 5 patients, *in vitro* studies, reviews, comments, letters, and studies on other locations of osteomyelitis. Studies in which >10% of patients did not have diabetes were excluded unless the results for these patients were presented separately. Only publications in the English language were included.

Parameters evaluated included healing rates, time, and complications, such as further surgical interventions, amputation rates, and mortality. Clinical presentation, laboratory investigation, radiological evaluation, antibiotic therapy, duration of symptoms, previous surgical procedures, and comorbidities were also recorded.

## 3 Results

In total, 16 studies were included (**Table 1**): 3 randomized controlled trials (RCTs) [15-17], 3 retrospective studies (RSs) [18-20], and 10 case series [3, 21-29]. Overall, 9 studies described the use of calcium sulphate tablets/beads, 4 studies assessed the use of gentamicin-impregnated collagen sponges, and one study used either calcium sulfate tablets or gentamicin-impregnated sponges. Four studies used PMMA for definitive treatment, and one study used PMMA as part of a two-stage procedure.

**Table 1.** Main findings from the included studies on local antibiotic treatment in foot infections and diabetic foot osteomyelitis (DFU)

Study Design	Design	Number of patients	Intervention	Follow-up	Results	Complications	
Lipsky *et al*., 2012	RCT	56 patients with moderately infected diabetic foot ulcers, randomized into treatment (n=38) and control (n=18) group.	Daily topical application of gentamicin-impregnated collagen sponges combined with systemic antibiotics compared with systemic antibiotic therapy alone. Standard diabetic wound management, including sharp surgical debridement at each visit.	14 to 28 days of treatment plus 2 weeks after treatment was discontinued.	The treatment group had a significantly higher proportion of patients with a clinical cure than the control group. Patients in the treatment group also had a higher rate of eradication of baseline pathogens and a reduced time to pathogen eradication.	The most common adverse events occurring in at least two patients per group were infections with skin ulcers, tinea pedis, and increased blood creatinine concentration.	
Varga *et al*., 2014	RCT	50 DFO patients were randomized into treatment (n=25) and control (n=25) group.	Gentamicin-impregnated collagen sponges peri-operatively in comparison with minor amputations without sponges. Systemic antibiotics were administered to both groups.	Indications for amputation were non-healing ulceration (more than 6 weeks)	There was no significant difference in hospital stay and further surgery between the groups. Wound healing duration in the treatment group was significantly better than in the control group.	Three re-amputations (1 major and 2 minor) were necessary for non-healing wounds in the treatment group. In the control group, 4 minor re-amputations were performed.	
Uckay *et al*., 2018	RCT	88 DFO patients were randomized into treatment (n=45) and control (n=43) group.	Gentamicin-impregnated collagen sponges with systemic antibiotic vs. systemic antibiotics alone; surgical debridement if there was a clinical need to remove necrosis or to drain an abscess.	14-28 days of treatment plus 10 days after treatment was discontinued.	73% showed total clinical cure, 15% significant improvement, and 52% showed total eradication of all pathogens. Regarding the final clinical cure, there was no difference in favor of the gentamicin-sponge.	There was a tendency towards more rapid healing in the gentamicin-sponge group. Gentamicin-sponges were very well tolerated, without any attributed adverse events.	
Krause *et al*., 2009	RS	65 cases (60 patients) of amputation for forefoot DFO were divided into beads (n=49) and control (n=16) group.	Application of tobramycin-impregnated calcium sulfate beads in addition to transmetatarsal amputation and standard treatment vs. no beads.	17 patients died and 3 were lost to follow-up after 29 months.	The beads group showed a lower rate for wound breakdown and further surgery, but there was no difference in length of hospital stay or rate of conversion to below-the-knee amputation.	27% in the beads group and 25% in the control group had to be converted to transtibial amputation.	
Qin *et al*., 2019	RS	48 limbs (46 patients) with DFO: 20 limbs (18 patients) were included in the calcium sulfate and 28 limbs (28 patients) in the control group.	Vancomycin and/or gentamicin-impregnated calcium sulfate beads after bone resection vs. bone resection alone. Systemic antibiotics in both groups.	At least 12 months	Local antibiotics prevented the recurrence of DFO, but did not improve the healing rate, reduce the postoperative amputation rate, or shorten time to healing.	Prolonged postoperative leakage in the CS group was the most common complication.	
Chatzipapas *et al*., 2020	RS	25 patients with forefoot and calcaneal DFO were divided into 3 groups: PMMA (n=9), H/CSF (n=8), and control (n=8).	Gentamicin-impregnated PMMA or H/CSF beads or nothing plus minor surgery. Concomitant antibiotics (first intravenously, later orally).	At least 12 months	All healing parameters were improved in both local antibiotic groups, but they did not reach statistical significance.	Recurrence of DFO in two patients, one in the PMMA group and one control. The latter underwent amputation.	
Krause *et al*., 2009	CS	16 patients (15 had diabetes) with forefoot full-thickness soft tissue defects.	Primary ulcer excision, surgical debridement, antibiotic-impregnated PMMA, and immobilization. 3 days later, 2^nd^ debridement, V-Y fasciocutaneous advancement flap coverage. Osseous defects were filled with either allogeneic bone graft impregnated with PRP or a permanent antibiotic-impregnated PMMA spacer.	15±9 months (range 4-34)	All but 4 flaps healed primarily, with each developing marginal dehiscence which healed with local wound care measures.	Two deep infections occurred despite the healing of the flap, which necessitated transmetatarsal amputation with split-thickness skin graft coverage.	
Gauland, 2011	CS	337 patients with lower extremity osteomyelitis.	Locally implanted vancomycin- and gentamicin-impregnated calcium sulfate tablets in the surgical debridement site.	Max of 5 years	279 of 323 patients were clinically healed without the use of intravenous antibiotics.	20 of 323 patients required amputation, 12 of which were digital amputations, 2 ray amputations, and 6 below-knee amputations.
Melamed and Peled, 2012	CS	23 cases of osteomyelitis and associated severe infection of forefoot joints in 20 consecutive patients.	Gentamicin/vancomycin-impregnated cement spacer placement and extensive meticulous debridement.	21±10 months	21 cases healed and two required toe amputation. The spacer was left permanently in 10 patients, removed with arthrodesis in six, and removed without arthrodesis in five.	One patient recovered, but subsequently underwent transtibial amputation due to infection of a different site.
Walsh and Yates, 2013	CS	10 patients (7 diabetes) with calcaneal osteomyelitis.	Calcanectomy followed by tobramycin-impregnated calcium sulfate or gentamicin-impregnated collagen locally.	Over 3 years	5/7 diabetes patients healed at a mean of 64 days.	2/7 diabetes patients required transtibial amputation after multiple debridements.
Jogia *et al*., 2015	CS	20 patients with forefoot DFO.	Minimal surgical intervention plus highly purified synthetic calcium sulfate impregnated with vancomycin and gentamicin locally	Over 18 months	All patients achieved healing with a median period of 5 weeks and no recurrence.	No adverse reactions.
Panagopoulos *et al*., 2015	CS	8 patients with chronic metatarsal and calcaneal DFO.	Gentamicin-impregnated PMMA or calcium sulfate/carbonate beads locally administrated. Concomitant antibiotics (first intravenously, later orally).	12 months	In all patients, DFO was successfully treated. Wound healing was seen in 6 patients.	One patient developed new ulceration in the ipsilateral and contralateral foot within 24 months.
Dalla Paola *et al*., 2015	CS	28 patients with forefoot DFO.	After surgical debridement with removal of the infected bone, vancomycin/ gentamycin-impregnated bone cement was inserted and the treated area was stabilized with an external fixator.	12±7 months	In 24 patients, no recurrence of ulceration and no transfer ulceration, shoe fit problems, or gait abnormalities were detected.	Four patients developed relapse of the ulceration. One of them underwent a percutaneous revascularization procedure and transmetatarsal amputation.
Elmarsafi *et a*l., 2017	CS	30 patients (27 with diabetes) with foot osteomyelitis.	PMMA and gentamicin/vancomycin-impregnated cement spacers placed into a previously infected foot after surgical excisional debridement.	Average 52 months; range 12 to 111 months	20 successful spacers. Of the remaining 10 patients, 8 underwent eventual ipsilateral partial foot amputation.	No major amputations had been required on the ipsilateral side. 3 patients underwent contralateral below-the-knee amputations.
Drampalos *et a*l., 2018	CS	12 patients with chronic calcaneal osteomyelitis.	A gentamycin-impregnated synthetic mixture of calcium sulfate and hydroxyapatite injected in multiple tunnels. Systemic antibiotics for 8-12 weeks.	Average 16 weeks; range 12- 18 months	Infection was eradicated and the wound healed in all 12 patients with a single-stage procedure. In 6 patients, the wound was closed primarily.	VAC needed in 6 patients; one underwent a reverse sural flap procedure at a second stage.
Niazi *et al*., 2019	CS	70 DFO patients	Gentamicin-impregnated calcium sulfate/hydroxyapatite bio-composite along with surgical debridement and systemic antibiotics.	Average 10 months; range 4-28 months	Infection was eradicated in 63 patients with an average time to ulcer healing of 12 weeks. No additional recurrence of infection was seen in any patient and no local or systemic side effects presented in any patients during treatment.	Seven patients were not cured and required further treatment. Five patients had a below-knee amputation.

**Legend:** CS - case study series, DFO - diabetic foot osteomyelitis, H/CSF - hydroxyapatite and calcium sulfate, PMMA - polymethylmethacrylate, PRP - platelet-rich plasma, RCT - randomized controlled trial, RS - retrospective study, VAC - vacuum assistance.

### 3.1 Randomized controlled trials

Lipsky *et al*. randomized 56 patients with moderately infected diabetic foot ulcers in a 2:1 ratio into 2 groups, one with and the other without the use of a gentamicin-collagen sponge in addition to standard care for up to 28 days [15]. Significantly higher rates of clinical cure and eradication of pathogens were achieved in the gentamicin-collagen sponge group [15]. Safety data were similar between the 2 groups.

Varga *et al*. investigated the efficacy of a gentamicin-collagen sponge application into wounds after minor amputation for non-healing ulcers with DFO [16]. Fifty diabetes patients were randomized to the add-on gentamicin sponge or usual care group. All patients received systemic antibiotics according to their antibiogram profile. In the gentamicin sponge group, wound healing duration was significantly shortened by almost 2 weeks. No differences were observed between the groups in length of hospitalization or number of revisions for wound breakdown or subsequent amputations.

Uçkay *et al*. continued their initial trial re-examining the potential benefits of gentamicin-collagen sponges in a larger RTC of 88 patients with DFO and prolonged follow-up [17]. There was no difference in clinical cure rates in favor of the gentamicin-sponge. However, a small trend towards faster healing was noted in the gentamicin-collagen sponge group. Similar to the other studies, local antibiotic delivery was not associated with safety concerns.

### 3.2 Retrospective studies

Krause *et al*. assessed the effect of local tobramycinimpregnated calcium sulfate beads in addition to standard treatment after transmetatarsal amputation in diabetes patients with non-healing forefoot full-thickness ulceration with DFO or skin necrosis [18]. In total, data from 65 amputations were reviewed, including 49 cases in the beads group and 16 cases in the group without beads. Wound breakdown rates were significantly lower in the beads group. In this group, there was also a non-significant reduction in time of healing. There were no differences between the groups in length of hospitalization and need for ipsilateral second transtibial amputation.

Qin *et al*. compared infected bone resection combined with adjuvant antibiotic-impregnated calcium sulfate vs. infected bone resection alone for the treatment of DFO in 46 patients [19]. Antibiotic-impregnated calcium sulfate prevented the recurrence of DFO, but showed no significant improvement in healing rates, post-operative amputation rate, and time of healing.

In the most recent retrospective study, Chatzipapas *et al*. recruited 25 patients with forefoot and calcaneal DFO who were divided into 3 groups [20]. Healing rates were 100% in the PMMA group (surgical debridement in combination with the local application of antibiotic-loaded PMMA beads), 87.5% in the calcium sulfate group (surgical debridement in combination with the local application of hydroxyapatite and calcium sulfate beads), and 87.5% in the control group (surgical debridement only) [20].

### 3.3 Case series

Roukis *et al*. studied 16 patients (15 diabetes) with medical comorbidities and offered two-stage reconstruction with a surgical skin flap of full-thickness foot ulcers using a V-Y technique for patients with high risk of wound breakdown [21]. Antibiotic-impregnated PMMA spacers were used in all first-stage procedures. After 3 days, a second-stage procedure was performed, involving further debridement, V-Y flap cover, and filling of bone defects with either bone graft combined with platelet-rich plasma or with antibiotic-impregnated PMMA. This treatment resulted in primary healing in 9 of 15 patients and secondary healing with dressings in a further four. Two patients required transmetatarsal amputation at a mean follow-up of 15 months.

The large case series by Gauland evaluated vancomycin- and gentamicin-loaded calcium sulfate tablets for lower-extremity osteomyelitis in 337 patients [22] . Damaged bone and soft tissues were resected and calcium sulfate tablets were inserted in the dead space. Overall, 86.4% of patients were treated without intravenous antibiotics and 7.4% with intravenous antibiotic administration. The remaining 6.2% was treated with amputation. Furthermore, 70% of patients healed even without oral antibiotic administration.

Melamed and Peled investigated the use of an antibiotic-impregnated cement spacer in 23 cases of forefoot DFO [23]. Of the 23 cases treated by meticulous debridement and antibiotic-impregnated cement spacer implantation, 21 healed successfully, while the spacer was left permanently in 10 patients. Transfer lesions occurred in one patient only. In two patients, it was necessary to amputate the affected part that did not heal [23].

Walsh and Yates reported that 5 out of 7 Wagner grade 3 ulcers healed with calcanectomy plus gentamicin-impregnated collagen sponge or calcium sulfate with tobramycin [24]. Mean healing time was 64 days (range not stated).

Dalla Paola *et al*. used vancomycin- and gentamycin-loaded bone cement after surgical debridement with removal of the infected bone for first metatarsophalangeal DFO [25]. They reported healing in 24 of 28 patients without new ulceration, shoe fit problems, or gait abnormalities. Four patients developed a relapse of the ulceration and one of them underwent percutaneous revascularization and transmetatarsal amputation.

Jogia *et al*. reported 100% cure in 20 DFO patients who had failed to respond to routine wound debridement, systemic antibiotics, and offloading [26]. Their approach included excision of bone sequestrate and application of biodegradable highly purified synthetic calcium sulfate pellets containing vancomycin and gentamicin. Postoperative systemic antibiotic treatment was decided on an individual basis.

Panagopoulos *et al*. included 8 patients with chronic metatarsal or calcaneal DFO [3]. These patients were successfully treated with gentamicin delivery either with PMMA cement beads or bone graft substitutes. Local antibiotics were applied after minor surgery in combination with systemic antibiotics. Gentamycin beads were absorbed in ≤2 months without surgical removal. Wound healing was seen in 6 patients.

Elmarsafi *et al*. evaluated 27 DFO patients treated by PMMA and gentamicin/vancomycin-eluting cement spacers after surgical excisional debridement [27]. Among these patients, 20 spacers were successfully retained or exchanged. Of the 10 patients requiring spacer removal, 4 underwent removal with subsequent arthrodesis and 6 with subsequent pseudoarthrosis, while 8 required ipsilateral partial foot amputation not related to spacer use or removal.

Drampalos *et al*. successfully treated 12 consecutive patients with calcaneal DFO using bone debridement and local delivery in drilled tunnels of a gentamycin-loaded absorbable calcium sulfate/hydroxyapatite biocomposite [28]. One patient required a subsequent flap operation and 6 needed vacuum-assisted closure. There was also one case of prolonged wound leakage. No major amputation was required.

Niazi *et al*. evaluated 70 DFO patients treated by debridement, local antibiotic-loaded calcium sulfate/ hydroxyapatite bio-composite, and systemic antibiotics based on intra-operative cultures [29]. This treatment resulted in a healing rate of 90%, and there was no recurrence of infection.

## 4 Discussion and conclusions

DFO remains difficult to treat. Therefore, add-on local antibiotics have been attempted post-operatively to improve outcomes [30]. However, definitive supportive evidence is still rare. In RCTs, gentamicinimpregnated collagen sponge significantly improved clinical cure rates and slightly improved duration of hospitalization. In RSs, antibiotic-impregnated calcium sulfate beads non-significantly improved all healing parameters, but did not reduce post-operative amputation rates or time to healing. The majority of case series used calcium sulfate beads, achieving adequate rates of healing and eradication of infection.

Importantly, there are limitations in available evidence. In fact, only 3 RCTs were identified [15-17]. Further limitations include small patient numbers, wide range of inclusion criteria, heterogeneity of DFO, and the variety of surgical techniques. Accordingly, large rigorously designed RCTs with clear inclusion criteria and procedures are required to shed more light on this issue. Finally, it is still unclear how patients who would most benefit from this add-on therapy can be identified or selected.

In conclusion, add-on local antibiotic delivery following surgery for DFO has achieved some favorable results, mainly healing and eradication rates. Nonetheless, evidence is still limited, while methods and criteria used in the studies have been heterogeneous. Certainly, local antibiotic delivery represents an important step towards improved wound treatment, but more robust evidence is needed, especially on its efficacy in DFO. If its efficacy is finally confirmed, this therapeutic adjunct will certainly enrich our armamentarium for one of the most dangerous diabetic complications [31, 32].

## Abbreviations

CS: case study series

DFO: diabetic foot osteomyelitis

H/CSF: hydroxyapatite and calcium sulfate

PMMA: polymethylmethacrylate

PRP: platelet-rich plasma

RCT: randomized controlled trial

RS: retrospective study

VAC: vacuum-assisted closure
